# Dynamic white matter microstructure in anorexia nervosa: associations with neurofilament light and leptin across early weight restoration

**DOI:** 10.21203/rs.3.rs-9434305/v1

**Published:** 2026-06-11

**Authors:** Svea Königsmann, Fabio Bernardoni, E. Caitlin Lloyd, Clara M. Breier, Marlene Riedl, Fernando Fernandez-Aranda, Philip Gorwood, Annekatrin Locke, Roy Bockholt, Veit Rössner, Daniel Geisler, Stefan Ehrlich

**Affiliations:** 1 Translational Developmental Neuroscience Section, Division of Psychological and Social Medicine and Developmental Neuroscience, Faculty of Medicine, Technische Universität Dresden, Dresden, Germany; 2 Department of Psychiatry, Columbia University Irving Medical Center, New York, US; 3 New York State Psychiatric Institute, New York, US; 4 Clinical Psychology Department, University Hospital of Bellvitge and IDIBELL, Barcelona, Spain; 5 CIBER Physiopathology of Obesity and Nutrition (CIBERobn), Instituto de Salud Carlos III, Barcelona, Spain; 6 Department of Clinical Sciences, School of Medicine and Health Sciences, University of Barcelona, Barcelona, Spain; 7 Université Paris Cité, INSERM, UMR1266, F-75013 Paris, France; 8 GHU Paris Psychiatrie et Neurosciences, Hôpital Sainte-Anne (CMME), 100 rue de la santé, F-75014 Paris, France; 9 Department of Child and Adolescent Psychiatry and Psychotherapy, Faculty of Medicine, University Hospital Carl Gustav Carus, Technische Universität Dresden, Dresden, Germany; 10 Eating Disorder Research and Treatment Center, Department of Child and Adolescent Psychiatry and Psychotherapy, Faculty of Medicine, Technische Universität Dresden, Dresden, Germany; 11 Department of Psychotherapy and Psychosomatic Medicine, Faculty of Medicine, University Hospital Carl Gustav Carus, TU Dresden, Dresden, Germany

## Abstract

Longitudinal changes in white matter (WM) microstructure from acute underweight to early weight restoration in anorexia nervosa (AN), and their underlying mechanisms, remain largely unexplored. Thus, this study aims to examine the relationship between microstructural alterations across WM regions and neurofilament light (NF-L), a marker of axonal injury; brain-derived neurotrophic factor (BDNF), a neuroprotective molecule; and leptin, a neuroplasticity-promoting hormone, during acute underweight and following short-term weight restoration. Diffusion-weighted MRI scans from 101 predominantly adolescent female participants with acute AN before and after short-term weight restoration and from 147 female healthy participants were used. Alterations in microstructural WM integrity assessed by fractional anisotropy (FA) were tested using linear mixed-effects models across groups. Additional analyses were used to investigate relationships of FA with NF-L, BDNF, and leptin. We found a mixed, mostly elevated FA signature at the acutely underweight stage, followed by incomplete normalization with weight gain. Subsequent analyses identified that change in FA was positively associated with reductions in NF-L levels above and beyond the effect of weight gain in one WM tract. Leptin increase accompanying short-term weight restoration mediated the effect of weight gain on FA decrease across four WM tracts (estimated average causal mediation effects range: −0.0050 to −0.0026; confidence intervals within −0.01 to 0.0). The results suggest that a decrease in FA may indicate rehabilitation of WM integrity in acute AN. In addition to nutritional rehabilitation, the increase of leptin levels during weight gain may be relevant for the normalization of specific WM tracts.

## Introduction

Anorexia Nervosa (AN) is an eating disorder characterized by distorted body image, persistent restriction of energy intake, and often excessive exercise, leading to pathological underweight with associated endocrine changes and profound medical complications ^1,2^. AN typically manifests during adolescence, and as relapse rates are high ^3,4^, many experience a chronic illness course. AN also has one of the highest mortality rates among all psychiatric disorders, resulting from high rates of comorbid psychiatric disorders and physiological complications of undernutrition ^5^.

In acutely underweight patients with AN, reduced gray matter volume and cortical thinning ^6,7^ as well as region-specific volumetric alterations in subcortical structures, e.g. amygdala, hippocampus, and thalamus ^8–10^, have been observed. Most of these alterations appear largely reversible with weight restoration: increases in body mass index (BMI) are associated with increases in both cortical thickness and subcortical volume gains ^11,12^. By contrast, findings on white matter (WM) alterations in AN are heterogeneous, and WM volumetric changes tend to be subtle and less consistently observed ^13^. Accordingly, the WM literature in AN has focused on microstructural properties as measured by diffusion MRI. Here, we examined whether these microstructural properties change from acute underweight to short-term weight restoration and whether these changes are related to longitudinal alterations in blood-based protein markers.

Diffusion tensor imaging (DTI) characterizes WM microstructure by quantifying directional water diffusivity ^14^. Among these, fractional anisotropy (FA)—derived from the tensor eigenvalues and associated eigenvectors—is one of the most common scalar metrics interpreted as a marker of WM integrity ^15^. Altered diffusion metrics have been reported in the majority of WM tracts in the acute state of AN, particularly in the corpus callosum, corticospinal tract, cingulum, and fornix ^13^. Yet findings remain inconsistent, with studies reporting higher and lower FA in the same WM tracts ^16,17^ and others finding no significant differences between participants with AN and healthy controls (HC) ^18,19^. Notably, studies reporting regional higher FA in participants with AN have mostly included adolescents ^16,17,20–22^. Among these studies, only a few longitudinal studies with relatively modest sample sizes and again mixed results exist, including two which yielded evidence for partial normalization following weight restoration ^17,22^. However, interpreting FA (and other DTI metrics) is complicated by the presence of “free water,” which refers to water molecules that are not restricted or bound by cellular structures ^23^. Free water is likely elevated among underweight individuals with AN, partly due to the loss of gray matter and ventricular enlargement that occurs with severely low weight ^24^. This may result in the contamination of WM voxels that are close to fluid-filled spaces ^25^. To date, free-water effects have not been systematically modeled or controlled for across the AN DTI literature, which may contribute to variability across reported WM findings.

The neurobiological mechanisms underlying the heterogeneous findings of FA in AN have been speculated to include potential consequences of acute underweight and undernutrition such as WM microstructure remodeling, but concrete processes remain largely unknown. Initial insight into the mechanisms underlying WM integrity alterations (i.e. changes in axon diameter, fiber packing, myelination, and membrane permeability) in AN may be gained from examining peripheral markers of nerve damage (such as neurofilament light [NF-L]), and neural growth factors (e.g. brain-derived neurotrophic factor [BDNF]), or adipocyte-derived, neuroplasticity-promoting hormones (such as leptin) ^26^, all measurable in blood.

NF-L, a structural scaffolding protein, is enriched in large-caliber myelinated axons and critical for axonal radial growth ^27^. Following axonal damage, NF-L is released, leading to increased concentrations in the CSF and blood, which has driven interest in NF-L as a biomarker of neuro-axonal injury ^28,29^. Elevated NF-L has been associated with metabolic disturbances in axons and the presence of lesions in a range of neurological disorders ^30^. Findings in AN suggest increased NF-L concentrations in the acute underweight state followed by rapid normalizations after partial weight restoration ^31,32^. NF-L is also associated with lower cortical thickness in various regions ^33^. Whether NF-L alterations additionally indicate that the FA alterations in AN underlie axonal and astrocytic damage processes remains unclear. In a recent cross-sectional study, WM volume reductions as well as altered WM connectivity networks (higher FA) were associated with higher NF-L concentrations in acute underweight AN, indicating a potential link between FA and axonal damage processes ^21^.

In contrast, concentrations of BDNF, a neuroprotective molecule, are reduced among acutely underweight patients with AN ^34^ and increase after short-term weight restoration ^35^. In general, BDNF serves a critical neuroprotective role by promoting neuronal growth, plasticity differentiation, and survival ^36^. Impairments in BDNF signaling have been observed to negatively impact subcortical structure plasticity and function in health and neurodegenerative diseases ^37^. Converging evidence highlights a prominent role of the hippocampus, where BDNF supports plasticity and memory-related processes ^38–40^. In a recent longitudinal study, increasing BDNF levels suggest contributions to global hippocampal recovery following short-term weight restoration in AN ^41^. Whether alterations in BDNF mediate underweight-related microstructural WM changes in AN remains unknown.

Hypoleptinemia, i.e. suppressed peripheral leptin levels, is considered a key neuroendocrine hallmark of acute underweight AN and impacts multiple somatic systems (hypothalamic-pituitary-adrenal, -gonadal, and -thyroid axes) as well as psychological processes, such as drive for thinness, disorder-specific rumination, mood, and reward processing ^42–45^. Recently, studies have found links between local volumetric reductions in both the amygdala and thalamus and decreases in leptin concentrations above and beyond the degree of underweight ^9,10,46^, aligning with findings from a leptin-replacement intervention study indicating that leptin can have sustained effects on brain tissue composition ^47^. Following short-term weight restoration, leptin levels increase and may thus index recovery from the acute undernourished state, supporting its role as a state marker and biomarker in AN ^44^. In general, the known (extra-)hypothalamic effects of leptin include processes such as neuro- and synaptogenesis, axon growth, and dendritic remodeling ^48^, which suggest not only a link between leptin levels and local volumetric changes in subcortical structures but also changes in WM integrity, reflected by FA.

To clarify potential mechanisms through which weight restoration relates to WM microstructure, we quantified the contribution of weight gain and tested whether longitudinal FA variation could also be explained by blood-based protein markers, over and above increases in weight. Specifically, given that NF-L is released following axonal damage, we examined whether FA changes mediate between weight gain and NF-L (i.e. whether a change in WM microstructure accompanies—and potentially explains—NF-L normalization). Conversely, we investigated whether changes in BDNF and/or leptin mediate potential FA changes during recovery, consistent with the notion that these signals may influence WM microstructure over and above weight-gain-related effects. In the present study, microstructural changes (reflected by FA) were identified using the ENIGMA DTI protocol, which included region of interest (ROI)-based tract-based spatial statistics (TBSS) and free-water correction to address ROIs vulnerable to partial volume effects. Extending previous TBSS studies, we aimed to explore mechanisms underlying potential weight-gain-related FA alterations in AN by examining associations with NF-L, BDNF, and leptin. Finally, we explored (A) whether potential longitudinal alterations in NF-L concentrations following weight gain are mediated by possible changes in FA (as explained above), and (B) whether potential alterations of BDNF and/or leptin mediate between weight gain and possible FA changes in AN. Such insights could deepen our understanding of the pathophysiological mechanisms underlying AN-related microstructural alterations and inform future therapeutic approaches, including experimental leptin supplementation.

## Methods

### Sample

The final study sample after data quality control (Supplementary Methods 1.1) consisted of 248 female participants (12–28 years old; n=101 acutely underweight patients with AN and 147 HC) selected from a total of 256 female participants (n=105 AN and 151 HC). Within the AN group, 74 of 101 participants were reassessed after short-term weight restoration (AN-TP2; all participants with AN in the present sample had a body mass index (BMI) increase ≥14.1%). AN was diagnosed by trained master’s/doctoral level research assistants in a face-to-face interview using the Structured Interview for Anorexia and Bulimia Nervosa (SIAB-EX; Fichter & Quadflieg, 2001) adapted to DSM-5 criteria ^1^. The sample partially overlaps with our previous studies ^17,19^ (Supplementary Methods 1.2).

Participants with AN were admitted to treatment programs at the University Hospital Dresden and assessed in the acutely underweight state (AN-TP1) within 96 h of beginning nutritional rehabilitation. Inclusion criteria for participants with AN included a BMI <10^th^ age percentile or <17.5 kg/m^2^ (if 15.5 years or older) and no sustained recent weight gain (>2.5 kg within the past four weeks or 2 kg within the past two weeks). HC participants had to have a current BMI of >10^th^ age percentile or 18.5–28 kg/m^2^ (if >15.5 years), be eumenorrhoeic, have no lifetime BMI <10^th^ age percentile or <17.5 kg/m^2^ (if >15.5 years), and have no history of psychiatric illness as assessed with the Mini International Neuropsychiatric Interview ^50,51^. Several exclusion criteria were applied to both groups including psychotropic medication within the past four weeks (except for selective serotonin reuptake inhibitors [SSRIs] and mirtazapine in AN; details in legend of Table 1) and medical conditions that influence eating behavior or body weight. Additional exclusion criteria for both groups included history of organic brain syndrome, schizophrenia, bipolar disorder, psychosis not otherwise specified, current inflammatory, neurologic, or metabolic disorders. Further, participants were not recruited if they were pregnant or breast feeding, had anemia, or if their estimated intelligence quotient (IQ) was <85.

The study was approved by the ethics committee of the Technische Universität Dresden (protocol number EK 14012011/EK 39022012) and carried out in accordance with the latest version of the Declaration of Helsinki. All participants (and the legal guardians of underage participants) gave written informed consent.

### Clinical measures

Participants completed German versions of the Eating Disorders Inventory-2 (EDI-2) ^52^ and core ED-specific symptoms were assessed by averaging EDI-2 subscales drive for thinness, body dissatisfaction, and bulimia ^31^. Depressive symptoms were examined using the Beck Depression Inventory-II (BDI-II) ^53^. IQ was estimated using age-appropriate versions of the Wechsler Intelligence Scales (Supplementary Methods 1.3). BMI standard deviation scores (BMI-SDS) were calculated for each participant to adjust for age and sex ^54,55^.

### Blood sampling and analysis

Blood sampling and MRI scanning took place between 7:45 and 9 a.m. as in our previous studies after an overnight fast ^31,56,57^.

Determination of NF-L levels were carried out as previously described ^31,58^ using the digital Simoa^™^ Human Neurology 4-Plex A assay in combination with the Simoa^™^ HD-1 Analyzer or the digital Simoa^®^ NF-light^™^ Advantage Kit in combination with the Simoa^™^ HD-X Analyzer (all Quanterix, Lexington, MA, USA). Serum NF-L samples were available from 77 AN-TP1, 65 AN-TP2, and 138 HC.

Serum free BDNF and plasma leptin were measured using a commercially available enzyme-linked immunosorbent assays (ELISA; BDNF: R&D Systems, Minneapolis/MN/USA; leptin: BioVendor Research and Diagnostic Products, Brno/Czech Republic). Serum free BDNF samples were available from 70 AN-TP1, 50 AN-TP2, and 132 HC. Left-censored leptin concentrations below the lower limit of detection (LOD) of the applied leptin assay (LOD=0.20 ng/mL; n=20 AN-TP1, 0 AN-TP2, and 0 HC) were imputed using a quantile regression multiple imputation approach for left-censored missing data (QRILC). Missing/unavailable leptin values (n=21 AN-TP1, 11 AN-TP2, and 10 HC) were not imputed (Supplementary Methods 1.4).

### Image acquisition

High-resolution three-dimensional T1-weighted (T1w) structural scans were acquired on a 3T scanner (Magnetom Trio, Siemens, Erlangen, Germany) using a rapid acquisition gradient echo (MP-RAGE) sequence with the same parameters as in our previous studies (Supplementary Methods 1.5) ^11,12^. Diffusion-weighted imaging (DWI) data were collected using a spin-echo sequence at 2.4 mm isotropic voxel resolution, 307×307×144 mm^3^ FoV, 128×128 matrix size, 60 slices, no inter-slice gap, TE=104 ms, TR=15 s, BW=2 056 Hz/Px, GRAPPA acceleration factor 2, 24 reference lines, and prescan normalize. A total of 32 diffusion sensitizing gradients (b=1 300 s/mm^2^) were applied and four images without diffusion weighting (b=0 s/mm^2^) were acquired.

### Processing of DWI

T1w images were visually checked for artifacts prior to image preprocessing as described in our previous studies ^11,12^. Imaging data used in the study were organized using the Brain Imaging Data Structure (BIDS) ^59^. Preprocessing of DWI data was performed using QSIPrep (v0.19.0; https://qsiprep.readthedocs.io/) ^60^ at the participant analysis level. Session-specific T1w images were used as anatomical references (Supplementary Methods 1.6) and each diffusion series was preprocessed separately. Diffusion preprocessing included denoising, correction for eddy-current-related distortions, correction for head motion, corresponding updates to diffusion gradient orientations, and resampling into a consistent space for subsequent analyses. No fieldmap-driven susceptibility distortion correction was applied.

DWI data were further processed using the Freewater estimatoR using iNtErpolated initialization (FERNET; https://github.com/DiCIPHR-Lab/Fernet/) ^61^ method in combination with FMRIB Software Library (FSL; v6.0.7.8) ^62^ utilities. This processing pipeline included estimation of the tissue volume fraction (i.e. free-water correction), extraction of a mean b0 brain mask, diffusion tensor fitting, and calculation of corresponding tensor metrics. The pipeline was also applied without free-water correction as a supplemental analysis (Supplementary Methods 1.6).

A TBSS ^63^ analysis was subsequently performed using the generalized TBSS pipeline developed by Billah ^64^. FA images were aligned to the MNI ENIGMA-provided template space using nonlinear registration. Individual FA values were projected onto a standardized WM skeleton thresholded at FA>0.2. ROI-based analysis was conducted by extracting individual mean FA values of 48 WM tracts as defined by the John Hopkins University atlas (Supplementary Methods 1.6). FA values exceeding three standard deviations (SD) from the mean of the respective study group were identified and, rather than excluded, were winsorized within each study group, preserving the overall data distribution ^65,66^. Quality assurance included visual inspection of FA maps both prior to and following TBSS analysis to identify misalignment (Supplementary Methods 1.1).

### Statistical analyses

NF-L, BDNF, and leptin concentration values were logarithmically transformed (log_10_-transformation) to reduce deviations from normality. As recommended by previous publications ^41,56^ and literature ^67^, BDNF values were adjusted for batch effects and storage time in each sample separately. Extreme outliers (>3 SD from the mean of the respective diagnostic group and timepoint after logarithmization and, for BDNF values only, residualization) were excluded prior to analysis (Supplementary Methods 1.7). Group comparisons of clinical, demographic, and blood-based protein marker levels were tested using *t*-tests: Longitudinal changes in the AN group (n=74 patients at both TP1 and TP2) were assessed using paired *t*-tests; group comparisons between the AN and HC samples (n=147 HC) were conducted using two-sample (unpaired) *t*-tests.

For all 48 ROIs, statistical modeling of FA across groups (contrasts of interest: AN-TP1–HC, AN-TP2–AN-TP1, and AN-TP2–HC) was conducted using participant-specific random-intercept linear mixed-effects (LME) models, adapted from our previous studies ^8,9,11,12^. The main LME model is defined as:

(1) $$FA={\text{Intercept}}_{\text{Random}}+\Delta_{AN}+A_{AN}*\left(BMI-{SDS}_{TP(x)}-BMI-SDSS_{TP2}\right)+B*Age+{BB}^{*}{Age}^{2⊥}$$

where the longitudinal differences in individual mean FA were modeled as a linear function of ΔBMI-SDS across short-term weight restoration (Supplementary Methods 1.7).

**Figure 1:**
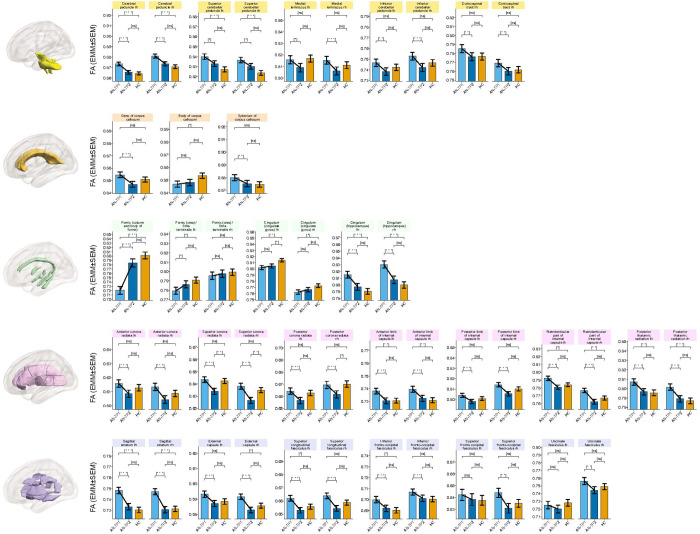
Cross-sectional and longitudinal group differences in FA Cross-sectional group effects (AN-TP1–HC and AN-TP2–HC) and longitudinal change AN-TP2–AN-TP1 (as a linear function of BMI-SDS increase) on FA in various WM tracts assessed through our main LME model covarying for linear and quadratic age effects. Regions associated with tracts in the brainstem, corpus callosum, limbic fibers, projection fibers, and association fibers are highlighted in yellow, orange, light green, pink, and purple, respectively. WM tracts were visualized using BrainNet Viewer (http://www.nitrc.org/projects/bnv/) ^83^. Bar plots display LME model estimates: estimated marginal mean (EMM) ± standard error of the mean (SEM) of individual FA values in each study group. Significant (*q*<0.05) pairwise contrasts are stated following FDR adjustment of *p*-values ^69^ across all contrasts and all WM tract ROIs. All inferential contrast tests derived from the same underlying main LME models, including contrast estimate (β; difference in adjusted marginal means), standard error (SE), *t*-statistic, 95% confidence interval (CI), unadjusted *p*-value, FDR-*q*, and random intercept variance (between-participant variability) as well as standard deviation (SD), are shown in Supplementary Table 2. [ns], nonsignificant; [*], *q*<0.05; [**], *q*<0.01; [***], *q*<0.001. Abbreviations: AN, anorexia nervosa; AN-TP1, participants with acute AN in the acutely underweight state; AN-TP2, participants with AN reassessed after short-term weight restoration; FA, fractional anisotropy; HC, healthy control participants; lh, left hemisphere; rh, right hemisphere; TP, timepoint.

Since our analyses revealed significant changes following short-term weight restoration (AN-TP2–AN-TP1, Figure 1), we investigated whether these longitudinal effects were associated with changes in concentration scores of the blood-based protein markers NF-L, BDNF, and leptin above and beyond the effects of weight gain (ΔBMI-SDS). Therefore, in a follow-up analysis modified LME models were used to model mean FA following short-term weight restoration in AN as:

(2) $$FA={\text{Intercept}}_{\text{Random}}+A_{AN}*\left(BMI-{SDS}_{TP(x)}-BMI-{SDS}_{TP2}\right)+B_{AN}*\left({\text{Marker}}_{TP(x)}-{\text{Marker}}_{TP2}\right)+C*Age+CC*{Age}^{2⊥}$$

where (${\text{Marker}}_{TP(x)}-{\text{Marker}}_{TP2}$) represents the within-participant change in NF-L, BDNF, or leptin and the other terms are defined as in the main model.

**Figure 2:**
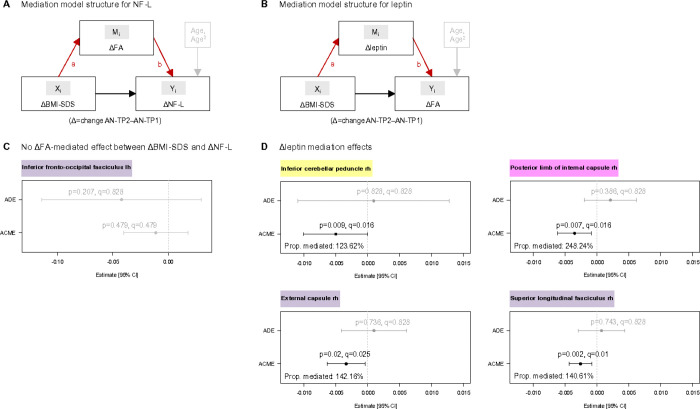
Mediation analyses in participants with AN for longitudinal change in WM ROIs following short-term weight restoration **A** Mediation model structure for NF-L (in participants with AN): weight gain (BMI-SDS increase) served as the independent variable/predictor (X_i_), change in FA as the mediator (M_i_), and change in NF-L levels, adjusted for linear and quadratic age effects, as the dependent variable/outcome (Y_i_). In multiple regression-based mediation analysis, mediation/indirect effect was computed as the product of coefficients of path *a* and *b* ^68^. **B** Mediation model structure for leptin (in participants with AN): weight gain (BMI-SDS increase) served as the independent variable/predictor (X_i_), change in leptin levels as the mediator (M_i_), and change in FA, adjusted for linear and quadratic age effects, as the dependent variable/outcome (Y_i_). In multiple regression-based mediation analysis, mediation/indirect effect was computed as the product of coefficients of path *a* and *b* ^68^. No mediation models were established for BDNF due to the absence of longitudinal associations with FA (Supplementary Table 5). **C** Results for the model in **A**, reporting the average direct effect (ADE) of weight gain on NF-L change scores after removing FA effects and the average causal mediation effect (ACME) through FA dynamics. **D** Results for the model in **B**, reporting ADEs of weight gain on FA dynamics after removing leptin effects and ACMEs through leptin change scores. Unstandardized estimates (points) and their 95% confidence intervals (CI; horizontal whiskers) are presented. To obtain robust CIs, nonparametric bootstrapping using the percentile method with 10 000 simulations was performed. Further, *p*-value and FDR-*q* (FDR-adjustment across all mediation models) are stated for ADE and ACME. Significant results are highlighted in black. Proportion (Prop.) mediated $\left(\frac{ACME}{ACME+ADE}\right)$ was computed when significant mediation occurred. Since the mediated proportion exceeded 100%, this reflects inconsistent mediation with opposing direct and indirect effects ^84^. In accordance with Imai et al. ^68^, sensitivity analyses of ACMEs through Δleptin were conducted. Results showed robustness of the indirect effect to a moderate unmeasured confounding in all four ROIs (Supplementary Results 2.3). Abbreviations: AN, anorexia nervosa; AN-TP1, participants with acute AN in the acutely underweight state; AN-TP2, participants with AN reassessed after short-term weight restoration; BMI-SDS, body mass index-standard deviation score; FA, fractional anisotropy; lh, left hemisphere; rh, right hemisphere; TP, timepoint.

Significant blood-based protein marker associations were followed-up with regression-based mediation models ^68^. These mediation models tested (A) whether longitudinal alterations of FA mediate between ΔBMI-SDS and alterations in blood-based NF-L concentrations, and (B) whether FA changes associated to ΔBMI-SDS changes were mediated by change in leptin concentrations (Supplementary Methods 1.7, Figure 2A, B).

Supplementary LME models used FA values obtained from standard preprocessed images, rather than free-water-corrected images to assess the influence of free water on fornix-related ROIs (Supplementary Methods 1.8). Regarding longitudinal associations between changes in concentrations of the blood-based protein markers and changes in FA, sensitivity analyses separately excluded participants with psychiatric comorbidities or SSRI/mirtazapine intake. Statistical significance was assessed at *α*=0.05. For all main and follow-up analyses, we corrected for multiple comparisons using a false discovery rate (FDR) ^69^ of *q*<0.05 across all contrasts of interest and all WM tract ROIs. All statistical analyses were conducted in R (v4.3.1) ^70^.

## Results

### Study sample and clinical measures

Sample characteristics are summarized in Table 1. AN were younger and had a higher IQ estimate, compared to HC. BMI(-SDS) increased but did not completely normalize in participants with AN following short-term weight restoration (treatment duration: mean±SD=90.27±33.48 days, range_min–max_=35–224 days; ΔBMI: mean±SD=27.95±10.1%, range_min–max_=14.1–62.9%). After this timeframe, depressive and ED symptoms decreased significantly. As expected, both symptoms were elevated in AN at the two timepoints, compared to HC. Changes in NF-L and leptin concentrations were observed. During weight restoration, NF-L concentration decreased significantly, whereas leptin concentration increased in patients with AN. At AN-TP1, NF-L concentrations were significantly higher and leptin concentrations significantly lower compared with HC. No differences were found between the 27 patients with AN who only participated at TP1 and the patients with AN of the longitudinal sample (Supplementary Results 2.1, Supplementary Table 1).

### FA alterations in relation to weight status in AN

At TP1, model estimates of FA were higher in AN compared to HC across 17 ROIs, including parts of the cerebral peduncle and internal capsule, hippocampus, post thalamic radiation, and several bilateral association fibers (Figure 1, Supplementary Table 2). By contrast, FA was lower in two ROIs related to the limbic system (fornix and left stria terminalis), the bilateral cingulate gyrus, and the body of the corpus callosum, while significant longitudinal increases were confined to the fornix and left stria terminalis.

From TP1 to TP2, FA decreased in 38 ROIs, notably within the corticospinal tract and superior longitudinal fasciculus, consistent with our previous findings ^17^.

At TP2, group differences between AN and HC were limited to 4 ROIs: the left cingulate gyrus and three sub-structures of the corona radiata.

Strikingly, the corona radiata sub-structures yielded lower FA values in AN at TP2 relative to both AN-TP1 and HC, whereas FA in the left cingulate gyrus was lower in AN across both timepoints compared to HC. In contrast, all ROIs that showed significant AN-TP1–HC and longitudinal FA differences did not differ between AN-TP2 and HC, indicating FA normalization in AN following short-term weight restoration in 19 ROIs.

Supplementary LME models using uncorrected FA values showed that both longitudinal and cross-sectional (AN-TP1–HC) FA differences in the column and body of fornix remained significant without free-water correction. By contrast, the left subcomponent of the fornix/stria terminalis showed no significant difference in uncorrected FA, in contrast to the main model, underscoring the sensitivity of FA to extracellular free water (Supplementary Results 2.2, Supplementary Table 3).

### Association of blood-based protein marker levels with FA

The 40 ROIs showing FA differences in AN between TP1 and TP2 were examined for associations with ΔNF-L, ΔBDNF, and Δleptin in longitudinal FA change (follow-up LME models). ΔBDNF did not predict longitudinal change in FA above or beyond the BMI-SDS effect. By contrast, decreasing NF-L and increasing leptin concentrations had an effect above and beyond the increasing BMI on change in FA in the left inferior fronto-occipital fasciculus and in four ROIs of the right hemisphere (external capsule, inferior cerebellar peduncle, posterior limb of internal capsule, and superior longitudinal fasciculus), respectively (Table 2, Supplementary Table 5). Additional ROIs showed uncorrected *p*<0.05 across all three blood-based protein markers, but did not survive FDR correction. Supplementary LME models excluding participants on SSRIs/mirtazapine (n=6) or with comorbidities (n=20) yielded results largely unchanged (Supplementary Results 2.3, Supplementary Table 6, 7).

### Leptin mediated weight-gain-related FA decrease in specific WM tracts

Based on our hypotheses, two mediation models were tested, one for each blood-based protein marker that showed longitudinal association with ΔFA beyond the effect of weight gain (Figure 2A, B). In the left inferior fronto-occipital fasciculus, neither the direct effect of ΔBMI-SDS on ΔNF-L nor the indirect effect via ΔFA was significant, providing no evidence for a FA-mediated effect between weight gain and decreasing NF-L (Figure 2C). By contrast, in a second model, increasing leptin significantly mediated the association between weight gain and FA decrease across all four ROIs: right external capsule (estimated average causal mediation effect [ACME]=−0.0034, 95% CI [−0.0063, −0.0004], *q*=0.025), right inferior cerebellar peduncle (ACME=−0.005, 95% CI [−0.01, 0.00], *q*=0.016), right posterior limb of internal capsule (ACME=−0.0035, 95% CI [−0.0062, −0.0009], *q*=0.016), and right superior longitudinal fasciculus (ACME=−0.0026, 95% CI [−0.0043, −0.0008], *q*=0.010). Direct effects of weight gain on FA were not significant in any ROI (Figure 2D).

## Discussion

The present longitudinal study integrates diffusion-weighted MRI with blood-based protein markers to probe mechanisms potentially underlying microstructural WM changes in a predominantly adolescent sample of participants with AN. We confirm a mixed—mostly elevated—FA signature at the acutely underweight stage, followed by broad, but incomplete, normalization with weight gain, using an ENIGMA-harmonized TBSS pipeline with free-water correction (part of ENIGMA AN workflow). Analyses regarding the longitudinal associations between FA and three different blood-based protein markers plus subsequent mediation analyses identified increases in body weight as a driver of FA decreases through increases in leptin concentrations in four out of 48 tracts. In a single WM tract, ΔNF-L were positively associated with ΔFA longitudinally, beyond the effect of increases in BMI-SDS in patients with AN. However, FA decreases did not mediate between weight-rehabilitation effects and decreases in NF-L levels. ΔBDNF did not predict ΔFA above and beyond the weight-restoration effect. These results suggest that WM alterations—reflected by FA—are state-related. Further, increases in leptin concentrations (i.e. endocrine recovery) seems to contribute to the normalization of specific WM tracts beyond the effect of weight gain early on in rehabilitation of patients with AN.

We initially compared FA across WM regions between acutely underweight patients with AN and HC, observing widespread group differences, consistent with prior findings ^16,71^. Following short-term weight restoration, FA decreased significantly in most ROIs in patients with AN, with increases limited to the fornix and left stria terminalis. Compared to HC, our findings suggest that young patients with AN exhibit widespread higher FA, which seems to be partially alleviated directly after short-term weight gain. These results, which might seem counterintuitive at first (since higher FA could be expected to indicate better WM integrity), but are consistent with other studies examining WM microstructure in AN ^16,17,20–22^. Another longitudinal study ^18^ reported no significant FA differences; however, this could be explained by the small sample size. Overall, a recent review by Leverett et al. ^13^ summarized longitudinal findings suggesting brain-wide FA increases in adolescents with acute AN that tend to normalize with weight rehabilitation. Notably, our findings regarding the fornix diverged from this broader pattern, showing increases rather than decreases in FA following short-term weight restoration. Since the fornix lies close to the ventricles, it is particularly susceptible to contamination by CSF due to loss of gray matter and ventricle enlargement in AN ^24^. Prior studies suggest that reduced FA in the fornix is an artifact of elevated free water complicating the interpretation of tensor metrics in this region. Although free-water correction increased FA in both AN and HC participants in this study (Supplementary Table 4), our free water-corrected findings still suggest significantly decreased FA in patients with AN relative to HC, in contrast to a prior study ^25^. Beyond free-water effects, discrepant FA findings across DTI studies may be partially attributable to the limited specificity of the single-tensor model in regions with complex fiber architecture (e.g. crossing fibers and variable fiber dispersion).

Although alterations in WM microstructure and connectivity have been studied before in AN, the underlying mechanisms, such as axonal and/or myelin sheath swelling, which could contribute to increased FA in AN ^72^, remain poorly understood. It has been hypothesized that microstructural brain alterations in AN may arise from ongoing neuroaxonal damage processes accompanied by morphological changes in neurons ^32,33^. In line with this hypothesis, we observed that decreases in NF-L—an axonal damage marker—were associated with reductions in FA in the left inferior fronto-occipital fasciculus (beyond the effects of BMI-SDS), suggesting that rehabilitation of axonal integrity may contribute to FA normalization during weight restoration ^28^. The absence of a ΔFA-mediated effect between weight gain and decreasing NF-L indicates that weight gain does not influence the associated normalization of both acutely elevated FA and NF-L. In contrast, although increases in BDNF have been suggested as a correlate of changes in hippocampal volume during weight restoration in AN ^41^, we found no significant associations with ΔFA beyond the effects of increases in BMI-SDS. This lack of association may be due to the fact that we did not find an increase in BDNF concentrations between TP1 and TP2, which we had anticipated ^34,40,41^. However, this absence of evidence for change aligns with a previous study ^56^, which also did not observe consistent change in BDNF following weight restoration in AN.

Building on evidence linking hypoleptinemia to subcortical volumetric reductions beyond increasing BMI-SDS effects ^8–10^, our longitudinal data extend these findings to WM microstructure by identifying Δleptin-mediated effects between weight gain and decreasing FA. Specifically, longitudinal decreases in FA were associated with increased leptin concentrations above and beyond effects of weight gain in the right hemisphere's external capsule, inferior cerebellar peduncle, posterior limb of internal capsule, and superior longitudinal fasciculus. Our analysis also demonstrated that leptin increases mediate the influence of short-term weight gain on FA decreases in all four ROIs. This pattern suggests that leptin may influence WM microstructure through pathways that are not fully captured by weight gain alone. Plausible biological mechanisms include leptin-related effects on activity-dependent plasticity ^26^, myelination, as well as glial maturation ^73^ and brain tissue composition ^47^, any of which could contribute to tract-specific changes in diffusion-derived indices. Thus, we speculate that in terms of translational potential leptin supplementation may help to reverse the increases in region-specific FA in AN in addition to nutritional rehabilitation. This interpretation is supported by findings identifying hypoleptinemia in acute AN as disease-specific neuroendocrine biomarker independent of BMI ^44,74^, and by studies reporting negative associations of leptin with WM integrity in schizophrenia and depression ^75,76^. Finally, a recent cross-sectional study of Hellerhoff et al. ^21^ hypothesized that lower leptin concentrations lead to axonal damage, and thus damaged WM connectivity, resulting in an increased release of NF-L in acute AN. Despite our longitudinal result of a positive association between NF-L decreases and ΔFA in the left inferior fronto-occipital fasciculus (beyond increasing BMI-SDS effects in patients with AN), we could not find evidence suggesting that weight gain influences this association between WM microstructure rehabilitation and NF-L normalization.

Our findings should be considered in light of some limitations. First, our results might not be generalizable beyond young female participants of predominantly European ancestry. Second, the study did not include an assessment of pubertal status. Although the majority of patients with AN were adolescent and age was controlled for in all statistical models, microstructural markers—including indices of restricted diffusion in WM—change dynamically across adolescence ^77^. Notably, endocrine–WM relationships in AN may vary by pubertal stage; prior work indicates a pubertal-stage-dependent association between higher ghrelin and lower fornix FA ^78^. Third, we observed a right-hemispheric dominance regarding associations between FA and leptin concentrations. However, left hemispheric regions showed uncorrected trends, which may indicate potential associations that could reach higher significance with a more large-scale study. Fourth, we only used single-shell diffusion MRI data instead of multi-shell data, which could have provided a more precise and reliable free-water-corrected FA ^79^. However, while prior studies have found that estimates of the free-water fraction may be poorly discriminated from tissue diffusion, the FERNET algorithm applied here uses an initialization technique that has been shown to provide more robust metrics compared to traditionally-used methods ^61^. Fifth, as with all diffusion tensor-based approaches, our FA measures are intrinsically limited in voxels with complex fiber architecture (e.g. crossing, fanning, or “kissing” fibers) and pronounced orientation dispersion. Consequently, not only FA but also other tensor-derived indices (axial, radial, and mean diffusivity) can be biased ^80^. Future work using more sophisticated diffusion methods (e.g. diffusion kurtosis imaging, neurite orientation dispersion and density imaging) and/or myelin-sensitive quantitative MRI may therefore yield more specific insights into the microstructural processes underlying the FA alterations observed in AN.

In conclusion, the current study identified predominantly elevated FA in acutely underweight patients with AN, followed by significant FA decreases across the majority of WM tracts after short-term weight restoration. Cross-sectional comparisons with HC further suggested partial normalization of FA after only a short period of weight gain. Mechanistically, longitudinal FA reductions during early treatment may reflect microstructural normalization. Since NF-L is released into CSF and blood in the context of neuro-axonal injury, decreasing NF-L levels concomitant with FA change are consistent with a treatment-related reduction in ongoing axonal damage processes, potentially related to alleviation of metabolic vulnerability of myelinated axons during renourishment ^30^. These findings may prompt further investigation of NF-L as a low-cost, minimally invasive marker to monitor neuro-axonal health during recovery. By linking plasma leptin (another accessible protein marker) with diffusion-derived indices, we further showed that increases in leptin mediate the association between weight gain and normalization of FA in several WM tracts. Together with previously reported associations between hypoleptinemia and reduced volumes of WM and subcortical structures, these results support a model in which the effects of weight gain on brain microstructural alterations are, at least in part, conveyed by the alleviation of hypoleptinemia. This could be explained by leptin-sensitive pathways relevant to WM development, including glial maturation and myelination ^73^. Emerging case-series evidence suggests potential therapeutic utility of exogenous leptin (metreleptin) in the treatment of AN ^81,82^. However, adequately powered longitudinal and interventional studies are needed to establish causal pathways and to determine whether targeting hypoleptinemia can beneficially modulate WM microstructure and clinical outcomes.

## Supplementary Material

This is a list of supplementary files associated with this preprint. Click to download.

• SupplementaryInformation.docx

## Figures and Tables

**Table 1:** Descriptive statistics and group comparisons summary of the study sample

	Acute AN group pre-/post-treatment^1^		Control Group	Contrast TP1–HC^3^		Contrast TP2–TP1^3^		Contrast TP2–HC^3^	
Characteristic	Mean ± SD (TP1)	Mean ± SD (TP2)	Mean ± SD	statistic	*q*	statistic	*q*	statistic	*q*

Age (years)	16.18 ± 2.58	16.43 ± 2.57	18.9 ± 4.3	−5.86	<0.001 ***	20.86	<0.001 ***	−5.33	<0.001 ***
IQ	114.88 ± 12.44	n/a	110.13 ± 9.58	2.8	0.007 **	n/a	n/a	n/a	n/a
BMI (kg/m^2^)	14.8 ± 1.31	18.83 ± 1.11	20.99 ± 2.34	−25.2	<0.001 ***	30.83	<0.001 ***	−9.31	<0.001 ***
BMI-SDS	−3.12 ± 1.14	−0.75 ± 0.65	−0.14 ± 0.7	−20.58	<0.001 ***	23.26	<0.001 ***	−6.34	<0.001 ***
Minimal lifetime BMI (kg/m^2^)	14.59 ± 1.36	n/a	19.67 ± 1.93	−22.32	<0.001 ***	n/a	n/a	n/a	n/a
EDI-2 core symptoms	25.54 ± 7.01	23.22 ± 7.69	15.66 ± 4.9	10.78	<0.001 ***	−3.43	0.001 **	7.56	<0.001 ***
BDI-II total	22.03 ± 10.82	12.9 ± 10.29	4.61 ± 4.89	13.18	<0.001 ***	−9.43	<0.001 ***	6.45	<0.001 ***

Blood-based markers	Median ± IQR (TP1)	Median ± IQR (TP2)	Median ± IQR						

NF-L (pg/mL)^2^	10.85 ± 7.81	6.09 ± 2.76	5.94 ± 2.87	7.69	<0.001 ***	−9.7	<0.001 ***	−0.09	0.931 (n.s.)
BDNF (ng/mL)^2^	20.63 ± 6.84	21.66 ± 7.38	20.58 ± 8.08	−1.01	0.36 (n.s.)	1.72	0.108 (n.s.)	0.23	0.852 (n.s.)
Leptin (ng/mL)^2^	0.58 ± 1.66	10.77 ± 9.61	10.32 ± 10.1	−13.19	<0.001 ***	13.99	<0.001 ***	−0.78	0.479 (n.s.)

AN, anorexia nervosa; AN-TP1, participants with acute AN in the acutely underweight state; AN-TP2, participants with AN reassessed after short-term weight restoration; BDI-II total, total score of Beck Depression Inventory-II; BDNF, brain-derived neurotrophic factor; BMI(-SDS), body mass index(-standard deviation score); EDI-2 core, averaged score comprising the core subscales drive for thinness, body dissatisfaction, and bulimia of Eating Disorder Inventory-2; HC, healthy control participants; IQ, intelligence quotient; IQR, Interquartile range; NF-L, neurofilament light; TP, timepoint; *p*-values were adjusted for multiple comparisons using the FDR procedure across all contrasts of interest within each variable, resulting in *q*-values being stated. (n.s.), nonsignificant

*, *q*<0.05

**, *q*<0.01

***, *q*<0.001.

1 Within the whole AN group, 91 of the participants were of the restrictive and 10 of the binge-eating/purging subtype; 14 were diagnosed with active comorbid psychiatric disorders (eight participants with depressive disorder, four with anxiety disorder, four with obsessive compulsive disorder, one with post-traumatic stress disorder, one with adaptation disorder, one with personality disorder, one with developmental disorder, and one with Tourette syndrome) at admission. SSRIs were taken by 3 AN-TP1 and 1 AN-TP2; mirtazapine by 1 AN-TP1 and 1 AN-TP2.

2 Blood-based protein marker concentrations were transformed to approximate normality: NF-L, BDNF, and leptin were log_10_-transformed. In case of significant batch and storage time effects, marker concentrations were subsequently residualized (Supplementary Methods 1.7). For better interpretability, median ± IQR of the raw blood-based protein marker values are displayed. However, group comparisons were computed with (residualized) log_10_-transformed marker values as described.

3 Group differences were tested using parametric *t*-tests: Longitudinal changes in the AN group (n=74 AN) were tested using paired *t*-tests; group comparisons between AN-TP1–HC and AN-TP2–HC (n=147 HC) were tested using Welch’s two-sample *t*-tests.

**Table 2:** Significant LME model results for longitudinal associations between Δmarker and change in FA following short-term weight restoration in AN

LME model:^1^ $FA={\text{Intercept}}_{\text{Random}}+A_{AN}*\left(BMI-{SDS}_{TP(x)}-BMI-{SDS}_{TP2}\right)+B_{AN}*\left({\text{Marker}}_{TP(x)}-{\text{Marker}}_{TP2}\right)+C*Age+CC*{Age}^{2⊥}$							
ROIs showing significant longitudinal FA differences (AN-TP2-AN-TP1)	ΔNF-L						
	β	SE	95% CI	*t*	*p*	*q*	*d*
Inferior fronto-occipital fasciculus lh	0.041	0.0117	0.3744 – 1.4538	3.5029	<0.001***	0.036*	0.92
	Δleptin						
	β	SE	95% CI	*t*	*p*	*q*	*d*
Inferior cerebellar peduncle rh	−0.0166	0.0055	−1.2375 – −0.2445	−3.0350	0.003**	0.034*	0.74
Posterior limb of internal capsule rh	−0.0089	0.0026	−1.4465 – −0.3590	−3.4301	0.001**	0.023*	0.91
External capsule rh	−0.0079	0.0024	−1.3426 – −0.3091	−3.2759	0.002**	0.023*	0.83
Superior longitudinal fasciculus rh	−0.0070	0.0020	−1.4843 – −0.3681	−3.4373	0.001**	0.023*	0.93

AN, anorexia nervosa; AN-TP1, participants with acute AN in the acutely underweight state; AN-TP2, participants with AN reassessed after short-term weight restoration; BMI(-SDS), body mass index(-standard deviation score); FA, fractional anisotropy; lh, left hemisphere; rh, right hemisphere; TP, timepoint; as model statistics for each blood-based protein biomarker, unstandardized coefficient (β), standard error (SE), 95% confidence interval (CI), *t*-value, unadjusted *p*-value, FDR-*q* (FDR-adjustment across all ROIs), and Cohen’s *d* are stated (only significant associations are shown; all results are shown in Supplementary Table 5).

*, *p/q*<0.05

**, *p/q*<0.01

***, *p/q*<0.001.

1 Associations of change scores in NF-L (log_10_-transformed), BDNF (both log_10_-transformed and adjusted for batch and storage time effects), and leptin (log_10_-transformed) with ΔFA were investigated following short-term weight restoration in AN (only those ROIs with significant change AN-TP2–AN-TP1 at FDR-*q*<0.05 according to the main LME model [Figure 1] were analyzed).
